# Differences in self-rated health and work ability between self-employed workers and employees: Results from a prospective cohort study in the Netherlands

**DOI:** 10.1371/journal.pone.0206618

**Published:** 2018-11-12

**Authors:** Ranu Sewdas, Sietske J. Tamminga, Cécile R. L. Boot, Swenne G. van den Heuvel, Angela G. de Boer, Allard J. van der Beek

**Affiliations:** 1 Amsterdam UMC, VU University Amsterdam, Department of Public and Occupational Health, Amsterdam Public Health Research Institute, Amsterdam, The Netherlands; 2 Amsterdam UMC, University of Amsterdam, Department Coronel Institute of Occupational Health, Amsterdam Public Health Research Institute, Amsterdam, The Netherlands; 3 Netherlands Organization for Applied Scientific Research TNO, Leiden, The Netherlands; Universtiy of Düsseldorf, GERMANY

## Abstract

**Background:**

With the increase of the statutory retirement age, the number of self-employed older workers will most likely increase. Therefore, this study aimed to explore: 1) the differences in self-rated health and work ability of self-employed workers and employees, 1) whether self-employment is associated with better self-rated health and work ability across three years, than employment, and 3) the role of sociodemographic, health- and work-related characteristics (e.g., mental load, physical load, and autonomy) in these relationships.

**Methods:**

Data was used from the Study on Transitions in Employment, Ability and Motivation, where self-employed (n = 1,029) and employees (n = 12,055) aged 45–64 years were followed during 2010–2013. Linear regression and generalized estimating equations analyses were carried out to study the differences in self-rated health and work ability (i.e., self-assessed work ability in relation to an individual’s resources and work demands) of self-employed and employees. To explore the role of sociodemographic, health-and work-related characteristics in these associations, we included interaction terms between these characteristics and employment status.

**Results:**

The self-employed had better work ability (8.3 versus 8.2), and better self-rated health (3.4 versus 3.3) than employees. Work ability of self-employed improved over time, compared to the changes over time in work ability among employees, but not no difference in change over time in self-rated health was found. None of the interaction terms were statistically significant (p>0.05).

**Conclusion:**

We observed higher scores in self-rated health and work ability among the self-employed than employees. Being self-employed leads to an increase in work ability across three years. The differences in work ability can be considered small, and more research is needed to establish the role of self-employment as a potential facilitator for sustainable employment.

## Background

In many European countries, a considerable proportion of the total working population is self-employed, for example, 16 per cent among European workers, and 17 per cent among Dutch workers [1, 2]. Although a large part, 58 per cent, of the self-employed are aged 25–49 years old, the self-employment rate of older workers is substantially higher compared to younger workers [3]. Namely, 39 per cent of the self-employed are aged 50–64 years old, compared to 3 per cent that are aged 15–24 years old [3]. Possible explanations for this are that independency is important at older age or that it is more difficult to obtain an employment contract at older age [4]. In addition, older workers tend to have higher expertise and probably a larger capital reserve compared to younger workers. With the increase of the statutory retirement age in many European countries, the labour market participation rate of older workers will be growing, and thereby, the number of self-employed older workers will most likely increase as well.

The self-employed differ from employees with regards to the working conditions. The self-employed have greater autonomy (i.e. job control), more flexibility in working hours and work pace compared to employees [2, 5]. These differences could explain the higher job satisfaction among the self-employed in comparison to employees [6, 7]. On the other hand, the self-employed are responsible for the survival of their business, while employees may not have this responsibility [8]. This responsibility could result in longer working hours, and longer working hours are associated with health problems [5, 9–11]. In line with this, prior research reports that the self-employed experience more work-related stress and more mental health problems than employees [12, 13]. As among the older working population serious health problems become increasingly common, it is of interest how differences in working conditions related to type of employment could influence the health and work ability of older workers.

In recent years, there has been an increasing interest in studying the effect of employment status on self-rated health and work ability among the total working population [10, 12, 14–16]. Work ability is defined as the self-assessment of a worker’s work ability in relation to an individual’s resources and work demands [17]. Studying these outcomes is important, as it is known that self-rated health and work ability are predictors of sickness absence and exit from paid work [18–20]. Previous studies showed that the self-employed are healthier and have a better work ability compared to employees [14–16].

However, it is unknown whether this also accounts for the older working population. Previous studies have shown the moderating role of certain socio-demographic factors (e.g. lower educational level and poor financial status), work characteristics (related to job control and job demands), and the presence of a chronic disease [21–24]. Therefore, in the present study, we will examine the relation between employment status and self-rated health and work ability among older workers, as well as the moderating role of socio-demographic factors, work characteristics and the presence of a chronic disease.

### Objectives

In this study, we aimed to explore among older workers: 1) the differences in self-rated health and work ability of self-employed workers and employees, 2) whether self-employment is associated to better self-rated health and work ability across three years, compared to employment, and 3) the role of sociodemographic, health- and work-related characteristics in these relationships.

## Methods

### Study population

The present study used a prospective design, and followed STROBE guidelines [25]. Data were used from the Study on Transitions in Employment, Ability and Motivation (STREAM), an existing prospective cohort study among a stratified sample of older workers aged 45+ years in the Netherlands [26]. Participants between 45 and 64 years in 2010 filled out an online questionnaire on various topics (among others, employment status, work characteristics and health) in the period 2010–2013, 2015, and 2016. In total 15,118 (71% of invited persons) respondents participated at baseline in 2010, of which approximately 12,000 participants were employees, 1,000 were self-employed workers, and 2,000 were non-employed adults. Further details on the STREAM study design can be found elsewhere [26]. In the present study, we selected employed and self-employed participants aged 45–64 years (n = 13,084) at baseline, and followed these participants during the period 2010–2013.

### Ethics approval

The Medical Ethical Committee of VU University Medical Center Amsterdam declared that the Medical Research Involving Human Subjects Act does not apply to STREAM. The Medical Ethical Committee had no objection to the execution of this study. In the information for participants that accompanied the online questionnaires, it was emphasized that the privacy of participants was guaranteed, all answers to the questions were treated confidentially, and all data were stored in secured computer systems. By filling in the questionnaire, participants implicitly gave permission for the use of the data. All data were fully anonymized before accessing the data.

### Dependent variables

Work ability was assessed using the first dimension of the Work Ability Index (WAI), in which workers estimate their current work ability compared to the lifetime best (0 not able to work– 10 lifetime best). Previous studies have shown that this single question item is highly associated with the overall WAI [27–30]. Self-rated health was assessed based on one-item about general perceived health status (1 poor– 5 excellent) [31]. Both outcomes were measured between 2010–2013, and both variables showed a normal distribution.

### Independent variable

The independent variable was self-reported employment status, operationalised as self-employed or employee (full time or part time). Participants who reported to be self-employed as well as employee, were grouped in the type of employment based on where they worked most hours.

### Covariates

The covariates were: level of education, financial situation, chronic disease, and work characteristics. Level of education was categorized as: 1) low (primary school, lower and intermediate secondary school or lower vocational training), 2) intermediate (higher secondary school or intermediate vocational training), and 3) high (higher vocational training, or university or higher). Financial situation was measured using the following question: ‘What is the financial situation of your household now?’. This variable was dichotomized into 0) those who said to have ‘some money left/a lot of money left’ and 1) those who said to be ‘very or somewhat short of money’. The presence of a chronic disease was determined by asking whether they had one of the following chronic diseases: hands, arms, legs, feet or back and neck symptoms; migraine or severe headache; cardiovascular diseases; asthma, bronchitis, emphysema; gastrointestinal disorders; diabetes; severe skin disease; psychological complaints; hearing problems; epilepsy; life-threatening disease (e.g. cancer); problems with vision; or other longstanding diseases. This variable was dichotomized into 0) those who answered ‘none’, and 1) those who had at least one of the listed chronic diseases.

The work characteristics were: mental load (high vs. low), physical load (high vs. low), and autonomy (low vs. high). Mental load was assessed using three items based on whether work activities require intensive thinking, thoughts and attention [32]. Physical workload was assessed using five items based on force exertion, static load and vibration [33]. Autonomy was assessed using five items on an individual’s opportunity to make decisions, decide the order and speed of performing tasks, find solutions, and take time off [34, 35]. Mental load, physical load, and autonomy were dichotomized at the median value. Potential confounders were gender (men/women) and age (continuous in years), and combined employment status (i.e. participants who reported to be self-employed as well as employee).

### Data analysis

Descriptive statistics (e.g. means, standard deviations, frequencies and percentages) were used to report baseline characteristics of the self-employed and employees in 2010–2013. Independent t-test and Pearson Chi-square test were used to report differences between self-employed and employees (significance at p<0.05).

Linear regression analyses were performed to study the differences in self-rated health and work ability of self-employed and employees using only information from baseline data in 2010, adjusted for age, sex, combined employment status, educational level, chronic disease, financial situation, mental load, and physical load.

Generalized estimating equations analyses (GEE) were carried out to study whether self-employment is related to better self-rated health and work ability across three years (2010–2013), compared to employment, adjusted for age, sex, combined employment status, educational level, chronic disease, financial situation, mental load, and physical load [36, 37]. The GEE model used in the present study was a time-lag model, implying that the repeated measurements of the independent variable (at T) studied were related to work ability or self-rated health reported at one measurement point later (at T+1). The independent variable and covariates were taken into account as time-dependent variables, except for age, sex, educational level, and financial situation. To capture changes between T and T+1 in the outcome measures, we adjusted for work ability or self-rated health at T, which is also known as an autoregressive technique [38, 39]. The ‘independent structure’ was used as the within-subject correlation structure, assuming that correlations between measurements are zero since the correlation already has been taken into account by adjusting for work ability or self-rated health at T [40, 41].

To explore the role of sociodemographic, health- and work-related characteristics for these relationships, interaction terms between low educational level, poor financial situation, presence of chronic disease, and poor work characteristics (i.e. high mental load, high physical load, and low autonomy) and the independent variable were separately added in the second step to the adjusted models. Multiplicative interaction was considered statistically significant if the interaction term showed a p-value below 0.05. For the categorical variable (i.e. educational level), it was considered statistically significant if the overall interaction term showed a p-value below 0.05.

In addition, additive interaction was studied. In general, additive interaction is relevant from a public health perspective; however, sometimes the multiplicative scale may be the one more naturally corresponding to biological mechanism[42, 43]. Therefore, when studying interaction, it is recommended to present both additive and multiplicative measures of interaction [42, 43]. In logistic regression models, additive interaction can be quantified using Relative Excess Risk due to Interaction (RERI) [43]. In the present study, however, linear regression analysis was used; additive interaction can be assessed by adding the covariate, the independent variable, and the interaction term in the model; the amount of additive interaction equals the regression coefficients of the interaction term [43]. If the additive interaction is not equal to zero, then an additive interaction is present; additive interactions can range from negative infinity (less than additivity) to positive infinity (more additivity). The confidence intervals around the interaction estimates were used to define significant interactions on an additive scale.

#### Sensitivity analyses

Using the same GEE model, a complete case analysis, and a subgroup analysis among individuals only with good self-rated health or good work ability at baseline were performed to study the relation between employment status and self-rated health or work ability across three years 2011–2013.

All statistical analyses were conducted with SPSS Statistics (version 22).

## Results

### Baseline characteristics

The baseline characteristics of the self-employed and employees are presented in Table 1. Among the self-employed, the percentage of men and of higher educated persons is higher than among employees. Furthermore, the self-employed report higher levels of autonomy, mental workload, and work ability in 2010–2013, compared to employees.

**Table 1:** Baseline characteristics of the self-employed and employees in 2010.

		Self-employed workers								Employees							
		2010 (n = 1029)		2011 (n = 834)		2012 (n = 800)		2013 (n = 765)		2010 (n = 12055)		2011 (n = 9373)		2012 (n = 8468)		2013 (n = 7281)	
Age, years	Mean (SD)	54.6	(5.8)	-		-		-		54.2	(5.5)	-		-		-	
Sex	Men	**655**	**(63.7%)**	-		-		-		**6782**	**(56.3%)**	-		-		-	
Educational level	Low	**242**	**(23.5%)**	-		-		-		**3280**	**(27.2%)**	-		-		-	
	Medium	400	(38.9%)	-		-		-		4682	(38.8%)	-		-		-	
	High	**387**	**(37.6%)**	-		-		-		**4093**	**(34.0%)**	-		-		-	
Financial situation	Difficulties	214	(20.9%)	-		-		-		2217	(18.5%)	-		-		-	
Work Ability, 0–10	Mean (SD)	**8.3**	**(1.2)**	**8.3**	**(1.1)**	**8.3**	**(1.1)**	**8.3**	**(1.2)**	**8.2**	**(1.2)**	**8.2**	**(1.2)**	**8.2**	**(1.2)**	**8.2**	**(1.2)**
Health status, 1–5	Mean (SD)	3.4	(0.9)	3.3	(0.9)	**3.3**	**(0.9)**	**3.4**	**(0.9)**	3.3	(0.9)	3.3	(0.9)	**3.3**	**(0.9)**	**3.3**	**(0.9)**
Chronic disease	Yes	589	(57.2%)	**443**	**(53.2%)**	**432**	**(54.3%)**	430	(57.2%)	7098	(58.9%)	**5838**	**(58.8%)**	**5724**	**(59.4%)**	5190	(57.9%)
Mental load	High	**578**	**(56.2%)**	**423**	**(54.1%)**	**373**	**(52.1%)**	**356**	**(54.2%)**	**6056**	**(50.3%)**	**4453**	**(48.3%)**	**4103**	**(49.2%)**	**3551**	**(49.3%)**
Physical load	High	490	(47.7%)	386	(49.6%)	355	(49.7%)	322	(49.1%)	5782	(48.1%)	4309	(46.8%)	3899	(46.9%)	3379	(47.0%)
Autonomy	Low	**173**	**(16.8%)**	**125**	**(16.0%)**	**138**	**(19.3%)**	**136**	**(20.7%)**	**5735**	**(47.6%)**	**4327**	**(46.9%)**	**3963**	**(47.5%)**	**3462**	**(48.1%)**

Bold values Pearson Chi-square test p-value < 0.05 or independent t-test p-value < 0.05

### The differences in self-rated health and work ability of self-employed and employees in 2010

The analyses showed that employment status was statistically significantly associated with self-rated health and work ability, indicating that the self-employed reported higher self-rated health and work ability than employees in 2010 (see Tables 2 and 3). No statistically significant additive or multiplicative interaction terms (p>0.05) were found between employment status and the covariates (e.g. educational level, poor financial situation, presence of chronic disease, and poor working conditions). Therefore, the association between employment status and work ability or self-rated health was not different among groups that differ on these covariates.

**Table 2:** Results of linear regression analyses on the cross-sectional relation between employment status (i.e. self-employed (n = 1,029) compared to employees (n = 12,055)) and self-rated health using data in 2010. And the results of interaction terms between risk factor and employment status (results from univariate models are presented).

Independent variable		Regression coefficients for health*^	95% confidence interval*^	
Employment status	Self-employed	**0.05**	**0.00–0.10**	—
**Covariates**^a^		Additive interaction estimates*	95% confidence interval for additive interaction estimates*	P-value for multiplicative interactions*
Educational level	High	Ref	Ref	0.91
	Medium	-0.04	-0.17–0.09	-
	Low	-0.02	-0.14–0.09	-
Chronic disease	Yes	-0.00	-0.10–0.10	0.98
Financial situation	Difficulties	-0.02	-0.14–0.10	0.75
Mental load	High	0.04	-0.06–0.14	0.41
Physical load	High	-0.06	-0.16–0.04	0.22
Autonomy	Low	0.04	-0.08–0.17	0.50

*adjusted for age, sex, combined employment status, educational level, chronic disease, financial situation, mental load, and physical load

^a^interaction term with employment status

Bold values statistically significant at p< 0.05

^a higher score indicates a better health status

**Table 3:** Results of linear regression analyses on the cross-sectional relation between employment status (i.e. self-employed (n = 1,029) compared to employees (n = 12,055)) and work ability using data in 2010. And the results of interaction terms between risk factor and employment status (results from univariate models are presented).

Independent variable		Regression coefficients for work ability*^	95% confidence interval *^	
Employment status	Self-employed	**0.14**	**0.07–0.22**	—
**Covariates**^a^		Additive interaction estimates*	95% confidence interval for additive interaction estimates*	P-value for multiplicative interactions*
Educational level	High	Ref	Ref	0.08
	Medium	**-0.18**	**-0.35–0.01**	**-**
	Low	-0.07	-0.27–0.13	-
Chronic disease	Yes	-0.03	-0.18–0.12	0.67
Financial situation	Difficulties	-0.13	-0.31–0.06	0.18
Mental load	High	0.09	-0.06–0.24	0.22
Physical load	High	-0.09	-0.24–0.06	0.23
Autonomy	Low	-0.04	-0.23–0.16	0.71

*adjusted for age, sex, combined employment status, educational level, chronic disease, financial situation, mental load, and physical load

^a^interaction term with employment status

Bold values statistically significant at p< 0.05

^a higher score indicates a higher work ability

### The relation between employment status and self-rated health or work ability across three years 2011–2013

Employment status was not associated with changes over time in self-rated health (see Table 4). Furthermore, the analysis revealed that self-employment was significantly associated with positive changes over time in work ability compared to the changes over time in work ability among employees (see Table 5). No statistically significant additive or multiplicative interaction terms (p>0.05) were found between self-employment and the covariates (e.g. educational level, poor financial situation, presence of chronic disease, and poor working conditions). Therefore, the association between employment status and changes over time in work ability or self-rated health in the period 2011–2013 was not different among groups that differ in these covariates.

**Table 4:** Regression coefficients, and confidence intervals estimated by GEE-analysis^a^ between employment status and changes in self-perceived health status over time, and the results of interaction terms between risk factor and employment status (results from univariate models are presented). Sample includes self-employed workers in 2010 (n = 1,029), 2011 (n = 834), 2012 (n = 800), and 2013 (n = 765) and employees in 2010 (n = 12,055), 2013 (n = 9,373), 2012 (n = 8,468), and 2013 (n = 7281).

Independent variable		Regression coefficients for health*^	95% confidence interval*^	
Employment status	Self-employed	0.02	0.01–0.04	-
**Covariates**^b^		Additive interaction estimates*	95% confidence interval for additive interaction estimates*	P-value for multiplicative interactions*
Educational level	High	Ref	Ref	0.31
	Medium	-0.03	-0.09–0.02	-
	Low	0.01	-0.06–0.08	-
Chronic disease	Yes	-0.03	-0.09–0.02	0.22
Financial situation	Difficulties	-0.00	-0.07–0.07	0.95
Mental load	High	0.03	-0.03–0.08	0.35
Physical load	High	-0.03	-0.08–0.02	0.27
Autonomy	Low	-0.03	-0.11–0.05	0.46

^a^ GEE-analysis with an independent correlation structure

^b^ interaction term with employment status

* adjusted for age, sex, combined employment status, educational level, chronic disease, financial situation, mental load, physical load, and self-perceived health status at T-1

^ a higher score indicates a better health status

**Table 5:** Regression coefficients, and confidence intervals estimated by GEE-analysis^a^ between employment status and changes in work ability over time, and the results of interaction terms between risk factor and employment status (results from univariate models are presented). Sample includes self-employed workers in 2010 (n = 1,029), 2011 (n = 834), 2012 (n = 800), and 2013 (n = 765) and employees in 2010 (n = 12,055), 2013 (n = 9,373), 2012 (n = 8,468), and 2013 (n = 7281).

Independent variable		Regression coefficients for work ability*^	95% confidence interval*^	
Employment status	Self-employed	**0.10**	**0.06–0.15**	—
**Covariates**^b^		Additive interaction estimates*	95% confidence interval for additive interaction estimates*	P-value for multiplicative interactions*
Educational level	High	Ref	Ref	0.42
	Medium	-0.04	-0.14–0.05	-
	Low	-0.04	-0.15–0.08	-
Chronic disease	Yes	-0.03	-0.12–0.05	0.44
Financial situation	Difficulties	-0.01	-0.12–0.11	0.91
Mental load	High	0.04	-0.05–0.13	0.38
Physical load	High	-0.06	-0.15–0.03	0.23
Autonomy	Low	-0.04	-0.15–0.07	0.52

^a^ GEE-analysis with an independent correlation structure

^b^ interaction term with employment status

* adjusted for age, sex, combined employment status, educational level, chronic disease, financial situation, mental load, physical load, and work ability score at T-1

Bold values statistically significant at p<0.05

^a higher score indicates a higher work ability

### Sensitivity analyses

The complete case analysis showed significant results for work ability (β 0.11; 95% CI: 0.06 to 0.16), but not for self-rated health (β 0.03; 95% CI: -0.00 to 0.05). Among individuals with good self-rated health at baseline, significant results were found for work ability (β 0.13; 95% CI: 0.06 to 0.19), but not for self-rated health (β 0.03; 95% CI: -0.02 to 0.08). Similarly, among individuals with good work ability at baseline, significant results were found for work ability (β 0.11; 95% CI: 0.07 to 0.16), but not for self-rated health (β 0.03; 95% CI: 0.00 to 0.05).

## Discussion

Firstly, we found that, among older workers, self-employed workers had better self-rated health and work ability compared to employees. Secondly, the work ability of self-employed workers statistically significantly improved over time compared to the changes in work ability among employees, while no difference over time was observed in self-rated health between the two groups. Thirdly, sociodemographic (e.g., level of education, financial situation), health (presence of a chronic disease) and work-related characteristics (e.g., mental load, physical load, and autonomy) did not modify these relationships.

We found evidence that self-employed workers had higher work ability scores, both at baseline as well as over time, than employees. This positive effect of being self-employed on work ability is explained by considerably higher levels of autonomy reported by self-employed workers in the present study, since our post hoc analysis (data from 2010) showed that adjustment for autonomy attenuated the relation between employment status and work ability with 68%. In line with previous studies, the present study showed that the self-employed have a better self-rated health compared to employees [14, 16]. A possible reason for a difference in self-rated health between self-employed workers and employees is a selection effect of healthier persons in self-employment. Another reason could be that the self-employed have more flexibility in working hours, and therefore, they are more likely to be involved in health promoting activities [10]. However, we did not find evidence that self-employment may lead to better self-rated health compared to employees across three years.

Our results with regard to the positive findings on work ability could be explained by two mechanisms: 1) older workers with better work ability are more likely to become self-employed, and 2) self-employment can positively influence the work ability of older workers leading to sustainable employment [16]. Since we do not know when and why the self-employed in our study started or made a transition into self-employment, we cannot provide evidence for the self-selection of older workers with better work ability into self-employment. However, in the sensitivity analysis, we included only older workers with good work ability at baseline to partly control for this self-selection. Since we still found that self-employed workers had better work ability compared to employees, we found evidence that self-employment can positively influence the work ability of older workers. However, the absolute differences in work ability (8.3 versus 8.1), and the standardised mean difference (0.2) can be considered a small difference [44]. This suggests that being self-employed has only a small positive effect on work ability over time.

Furthermore, it might be possible that in our study, the general population of workers (i.e., self-employed or employees) aged 45 years and older at baseline consists of relative healthy older workers for two reasons: (1) participation in paid work requires good health, especially at older ages, and (2) those in poor health have the tendency to leave employment [45]. This also called the ‘healthy worker effect’ and may explain why no interactions were found for those with low educational level, poor financial situation, presence of chronic disease, and poor work characteristics. It is likely that at baseline older workers with these characteristics do not differ in self-rated health or work ability between self-employed and employees. We recommend for further research to assess the older workers with a longer follow-up period to reduce the likelihood of health-related exit selection at baseline.

Strengths of this study include the availability of a large sample of self-employed and employees and the use of a follow-up period of three years. Some limitations should also be taken into consideration. Among the respondents with missing data, the self-employed workers had a higher work ability score compared to the employees (see S1 Table). However, the absolute differences (e.g., 8.3 versus 8.2) indicate that this difference was small. Furthermore, our results might reflect differences in certain work characteristics related to occupational groups across self-employed and employees, rather than differences in employment status. Another study found an indication that mortality rates were generally lower among self-employed workers compared to employees, but mortality rates varied across several industrial sectors [46]. However, we adjusted for several work characteristics, such as mental load, and physical load, which could be related to characteristics of different occupational groups.

The rapidly ageing workforce puts a pressure on policymakers to pay attention to keeping the workforce and especially the older workers healthy. Our results suggest that being self-employed might have a positive influence on work ability over time among older workers. Nevertheless, the difference in the work ability score is considered to be small. Further research is required to establish more definitive answers in the role of self-employment as a facilitator for sustainable employment. Furthermore, we underline the importance of taking into account a possible self-selection of older workers with better work ability into self-employment in future studies by identifying (health-related) factors related to the transition into self-employment.

Since good self-rated health and high work ability are important factors for employment, there is a need for interventions that are effective in stimulating a healthy and productive working life for older workers [47, 48]. Since we found only a small difference over time in work ability and no difference in self-rated health over time between self-employed and employees, our results suggest that these interventions are equally important for both groups. However, additional information is needed on how to implement these possible interventions among self-employment workers.

## Conclusion

We showed that the self-employed had better self-rated health and work ability than employees. Furthermore, self-employment was significantly associated with positive changes over time in work ability compared to the changes over time in work ability among employees. However, the difference in work ability was small, and more research is needed to establish the role of self-employment as a potential facilitator for sustainable employment.

## Supporting information

S1 Table Characteristics at baseline in 2010 for self-employed and employees with missing data. (DOCX)
