# Supplementary material for: Differences in self-rated health and work ability between self-employed workers and employees: Results from a prospective cohort study in the Netherlands
Source: PLoS One. 2018 Nov 12;13(11):e0206618. doi: 10.1371/journal.pone.0206618 (PMC6231617; doi:10.1371/journal.pone.0206618)
Supplement: S1 Table — (DOCX) [file pone.0206618.s001.docx]

**S1 Table. Characteristics at baseline in 2010 for self-employed and employees with missing data**

| **Characteristics** |  | **Self-employed workers (n=401)** | | **Employees (n=4162)** | |
| --- | --- | --- | --- | --- | --- |
| Age | mean (SD) | 54.0 | (5.7) | 53.5 | (5.5) |
| Sex | men | **271** | **(68%)** | **2341** | **(61%)** |
| Educational level | low | 104 | (26%) | 1130 | (27%) |
|  | medium | 167 | (42%) | 1673 | (40%) |
|  | high | 130 | (32%) | 1359 | (33%) |
| Health, 1-5 | mean (SD) | 3.3 | (0.9) | 3.3 | (0.9) |
| WAI, 1-10 | mean (SD) | **8.3** | **(1.2)** | **8.2** | **(1.2)** |
| Chronic disease | Yes | 231 | (58%) | 2424 | (58%) |
| Physical workload | High | 209 | (53%) | 2054 | (50%) |
| Mental workload | High | **229** | **(57%)** | **2063** | **(50%)** |
| Autonomy | Low | **75** | **(19%)** | **1943** | **(47%)** |
| Financial situation | Difficulties | 89 | (22%) | 845 | (20%) |

Bold values Pearson Chi-square test p-value < 0.05 or independent t-test p-value < 0.05
